# Manufacturing exosomes for wound healing: Comparative analysis of culture media

**DOI:** 10.1371/journal.pone.0313697

**Published:** 2024-11-14

**Authors:** Huy Hoang Dao, Thu-Huyen Nguyen, Diem Huong Hoang, Bach Duong Vu, Minh-Anh Tran, Mai Thi Le, Nhung Thi My Hoang, Anh Viet Bui, Uyen Thi Trang Than, Xuan-Hung Nguyen

**Affiliations:** 1 Vinmec Hi-Tech Center, Vinmec Healthcare System, Hanoi, Vietnam; 2 Faculty of Biology, VNU University of Science, Vietnam National University, Hanoi, Vietnam; 3 Vinmec-VinUni Institute of Immunology, Vinmec Healthcare System, Hanoi, Vietnam; 4 College of Health Sciences, VinUniversity, Hanoi, Vietnam; 5 University of Science and Technology of Hanoi, Vietnam Academy of Science and Technology, Hanoi, Vietnam; TotiCell Limited, Bangladesh, BANGLADESH

## Abstract

Mesenchymal stem cell (MSC)-derived exosomes (EXs) have emerged as promising therapeutic agents for wound healing. However, the optimal conditions for manufacturing MSC-derived EXs that maximize their wound-healing potential have yet to be established. Hence, we compared the efficacy of five different MSC culture media, including three different serum-free, a platelet-supplemented, and a fetal bovine serum-supplemented media, in exosome manufacturing for wound healing applications. Although umbilical cord-derived MSCs (UCMSCs) cultured in these media exhibited similar proliferation, morphology, MSC surface marker expression, and stemness, EXs derived from UCMSCs cultured in different culture media displayed varying levels of growth factors and cytokines. Notably, EXs derived from platelet-supplemented media (DM-PLT_EXs) exhibited significantly higher concentrations of keratinocyte growth factor (KGF), vascular endothelial growth factor (VEGF-A), platelet-derived growth factor (PDGF-BB), interleukin 6 (IL-6), interleukin 7 (IL-7), and interleukin 8 (IL-8) than EXs from other media. These differences correlated with the superior capability of DM-PLT_EXs to promote human skin fibroblast proliferation and stimulate angiogenesis of human umbilical vein endothelial cells, making them a more suitable choice for wound healing applications. Our findings emphasize the significance of the culture medium selection in tailoring the therapeutic potential of UCMSC-derived EXs for wound healing.

## Introduction

Wound healing is an intricate sequential process encompassing four overlapping phases of hemostasis, inflammation, proliferation, and tissue remodeling [1]. This process initiates promptly after an injury and entails coordinating various cell types, including immune cells, fibroblasts, and endothelial cells [1]. These cells also play a pivotal role in forming the extracellular matrix and secreting various factors, such as cytokines, chemokines, and growth factors, depending on different stages of the wound-healing process [1]. In developed countries, chronic wounds affect 1% to 2% of the population and consume 2% to 3% of healthcare budgets [2]. Thus, developing innovative technologies and products for wound treatments is crucial to alleviating chronic wound-associated societal burdens.

Cell therapy, including mesenchymal stem cells (MSCs), is emerging as an innovative method for regenerative medicine. MSCs spring from mesoderm during human embryonic development [3]. They possess the unique ability to self-renew and can differentiate into cell types crucial for wound healing, such as fibroblasts, under specific conditions [4]. In addition, the MSC secretome, comprising epidermal growth factor (EGF), fibroblast growth factor (FGF), insulin-like growth factor (IGF), platelet-derived growth factor (PDGF), transforming growth factor (TGF), and vascular endothelial growth factor (VEGF), has been shown to facilitate wound healing [5, 6]. These factors are encapsulated within the extracellular vesicles (EVs), making them promising cell-free candidates for replacing MSCs in clinical applications [7]. Compared to MSCs, MSC-derived EVs exhibit reduced immunogenicity, convenient storage, and remarkably potent biological activity. Hence, EVs, including exosomes, have been proposed as an alternative to conventional cell-based therapies for treating various diseases.

Exosomes (EXs) are lipid membrane-enclosed vesicles ranging in size from 40 to 250 nm. They are secreted by almost all cell types and contain a diverse array of bioactive molecules, such as mRNAs, microRNAs, lipids, and proteins [8–10]. These vesicles play an essential role in cell-to-cell communication by delivering packed biomolecules into the target cells via various pathways, such as phagocytosis, micropinocytosis, and clathrin-mediated endocytosis^9^. Moreover, MSC-derived EXs have promoted wound healing in pre-clinical and clinical trials. For instance, Ren et al. (2019) reported that various factors present in MSC-derived EXs, including PDGF, VEGF-A, EGF, and FGF, can stimulate angiogenesis [11]. These EXs have also been shown to support the proliferation and migration of epithelial cells [11]. Beyond their supportive roles in proliferation, migration, and angiogenesis, MSC-derived EXs have been found to reduce the inflammation response through their miR-223 and miR-181c [12, 13].

Despite showing great promise, expanding the MSCs for EV manufacturing and EV isolation remains a significant challenge to bring EVs into clinical applications. A typical MSC culture for EV production involves nutrient media supplemented with fetal bovine serum (FBS). However, the FBS-supplemented media raises concerns about introducing animal-derived substances into humans, potentially compromising the immune response to lipopolysaccharides or diseases [14, 15]. Moreover, materials derived from animals carry the risk of transmitting zoonotic viral or prion diseases to humans [16–18]. To mitigate these concerns, a xeno-free/serum-free medium has emerged as an alternative to FBS-supplemented media. To date, two categories of xeno-free/serum-free culture media have been applied to manufacture MSCs and EVs for clinical trials. The first type is the undefined culture medium, including media supplemented with lysed human blood-derived platelets (PLTs). PLT lysates could be harvested by freezing-thawing cycles of PLTs from blood banks [19] to release various growth factors, cytokines, and other regulatory molecules that support MSC proliferation [20]. The second category is the purely defined culture media, consisting of only defined synthetic and recombinant substances [19]. Nonetheless, a comprehensive, comparative analysis of these culture media on EX production, characteristics, and function, especially in wound healing settings, has not been done. Therefore, this study aims to evaluate the impact of five different cell culture media, including a conventional FBS supplemented, a PLT supplemented, and three serum-free defined media, on the characteristics and wound healing functions of UCMSC-derived EXs.

## Methodology

### Ethics declarations

For the umbilical cord collection from caesarean section, the study entitled “Manufacturing Exosomes for Wound Healing: Comparative Analysis of Culture Media” received approval from the Medical Ethics Committee of Vinmec Healthcare System (Date: 31.10.2022, No.119/2022/CN/HĐĐĐ VMEC) and was conducted following approved institutional guidelines. Donors signed written informed consent before donating their samples.

### Umbilical cord-derived mesenchymal stem cell expansion

Umbilical cords (UC) were collected from caesarean section between October 31, 2022, and May 31, 2023, with consent forms for UCMSC isolation (Ethical number: 119/2022/CN/HĐĐĐ VMEC). UCs were minced after washing and eradicating blood with phosphate buffer saline (PBS) (Thermo Fisher Scientific, USA) and 1% Penicillin/Streptomycin (Sigma-Aldrich, USA). Then, UC pieces were transferred into 50 mL falcon tubes with 500 U/mL of collagenase type I (Thermo Fisher Scientific, USA) and incubated for one or two hours for digestion at 37°C in the shaker. Subsequently, samples were diluted in PBS and centrifuged into cell pellets. Cells were then re-suspended with culture media and seeded with a density of 5000 cells / cm^2^ in culture flasks coated with CELLstart™ Substrate (ThermoFisher, USA) diluted in PBS (1:100) with a series of medium (Table 1). After reaching 80–90% confluency, the cells were split using CTS™ TrypLE™ Select Enzyme (ThermoFisher, USA) for the next passage.

**Table 1:** Three types of culture media for UC-MSC-derived EX manufacturing.

Group	Culture medium/condition	Abbreviation	Exosomes
Serum-free, defined culture media	STEMin1 medium (Himedia, USA)	Sin1	Sin1_EXs
	NutriStem medium (REproCELL, Belgium)	NutriS	NutriS_EXs
	StemMACS medium (Miltenyi Biotech, Germany)	SMACS	SMACS_EXs
PLT supplemented medium	DMEM/F12 (Thermo Fisher Scientific, USA) + 5% Platelet Lysate (PLT) (Sigma-Aldrich, USA)	DM-PLT	DM-PLT_EXs
Conventional FBS-supplemented medium	DMEM/F12 (Thermo Fisher Scientific, USA) + 10% EVs-depleted fetal bovine serum (Thermo Fisher Scientific, USA)	DM-FBS	DM-FBS_EXs

### Population doubling time

UCMSCs were examined for their proliferation rate through population doubling time in each passage. Population doubling time (PDT) was identified following the formula [21]:

$$PDT=\frac{Culture\;period\;in\;hours*ln\left(2\right)}{ln(number\;of\;cell\;T0)-lnln(number\;of\;cell\;T1)}$$

T0 is the seeding cell number, and T1 is the cell number at harvest. Cells were stained with trypan blue and calculated using the hemocytometer.

### UCMSC characterization

UCMSC surface markers were examined at passage three by flow cytometry using a Human MSC Analysis kit (BD Biosciences, USA). Cells were stained with positive control (anti-CD73 APC, anti-CD90 PerCP-Cy5.5, and anti-CD105 FITC) and negative cocktail control (anti-CD45 PE, anti-CD34 PE, anti-CD11b/CD19 PE, and HLA-DR PE). The results were captured and analyzed using a Beckman Coulter flow cytometer with Navios software 3.2.

### Fibroblast colony forming unit (CFU-F) assay

To determine the ability of UCMSCs to form fibroblast colony-forming units (CFU-F), UCMSCs at passage three were seeded at a density of 4 cells / cm^2^ in the 24-well plate in different culture media. The culture medium was replaced every three days and maintained for two weeks before the cells were fixed with absolute ethanol and stained with Giemsa 1X (Sigma-Aldrich, USA) at RT for 15 minutes. Then, cells were washed with distilled water to remove excess Giemsa, and the number of colonies was identified and quantified under a microscope.

### Tri-lineage differentiation

UCMSCs at passage three were seeded in 96-well plates to evaluate their tri-lineage differentiation capacity. After the initial culture of two days, the medium was replaced with an induction medium from StemPro™ Chondrogenesis Differentiation Kit (ThermoFisher, USA), StemPro™ Osteogenesis Differentiation Kit (ThermoFisher, USA), and StemPro™ Adipogenesis Differentiation Kit (ThermoFisher, USA) for chondrogenic, osteogenic, and adipogenic differentiation, respectively. For adipogenic differentiation, cells were maintained for 20 days before being fixed with 4% formaldehyde for 30 minutes and washed with PBS. Subsequently, cells were stained with a 0.5% Oil Red O (Sigma Aldrich, USA) diluted in distilled water at room temperature (RT) for 15 minutes and washed with PBS to observe the lipid-rich adipocytes. For osteogenic differentiation, UCMSCs were maintained for 25 days before being fixed with 4% formaldehyde for 30 minutes and washed with PBS. Then, the cells were stained with a 2% Alizarin Red S (Sigma Aldrich, USA) and rewashed twice with distilled water to visualize the mineralized matrix produced by osteoblasts. Regarding chondrogenic differentiation, UCMSCs were incubated with the chondrogenic differentiation medium for 22 days. Then, they were fixed with 4% formaldehyde for 30 minutes, washed with PBS, and stained with Alcian Blue (Sigma-Aldrich, USA) in 3% acetic acid. Finally, cells were washed with HCL 0.1N and distilled water twice. Images of differentiated cells were captured using an inverted microscope.

### Exosome isolation

The exosome isolation protocol followed the procedure detailed in our previous research [22]. Briefly, UCMSCs (P5) were seeded at 5000 cells / cm^2^ into T225 flasks (Nunc™ EasYFlask™ Cell Culture Flasks), then cells were maintained at 37°C and 5% CO2 without changing media in five different culture media until reaching 90% confluency. Subsequently, conditioned media was harvested for EXs isolating. Cell debris, apoptotic bodies, and microvesicles were removed by a centrifuge at 16,500 × *g* for 30 min at 4°C (Optima XPN-100 Ultracentrifuge, Beckman Coulter, California, USA). Then, these conditioned mediums were centrifuged at 100,000 × *g* for 90 minutes at 4°C to collect EX pellets. EX pellets were reconstituted in PBS and stored at -80°C for the downstream experiments.

Exosomes prepared for growth factor assay and functional assay were from the conditioned media of UCMSCs cultured in EV-depleted media (making up from EV-depleted FBS or PLT by a centrifugation at 120.000 x g for 18 hours to eliminate EVs).

### Transmission electron microscopy (TEM)

The diluted EXs were treated and labeled according to the procedure outlined in our prior research^22^. Briefly, exosome samples were fixed using 4% paraformaldehyde and subsequently placed onto Formvar-carbon coated grids (Ted Pella Inc., California, USA). The samples were washed eight times with PBS before being stained with uranyl-oxalate. The grids were then left to dry at room temperature. EXs were observed using a JEOL 1100 Transmission Electron Microscope (JEOL Ltd., Tokyo, Japan) at 80 kV at the National Institute of Hygiene and Epidemiology (NIHE).

### Western blot

Protein extraction and western blot were performed following what was described in the previous publication^22^. Briefly, total EX proteins were extracted by RIPA buffer (Thermo Fisher Scientific, USA) and then quantified by Pierce™ BCA Protein Assay Kit (Thermo Scientific, Massachusetts, USA). 15 μg exosomal proteins were loaded into each lane in 4–12% NuPAGE gels (Invitrogen, Massachusetts, USA) and underwent electrophoresis. Then, protein bands were transferred onto the membrane before being probed with primary antibodies (Abcam, UK) against CD9 and GAPDH, followed by incubating with goat anti-Rabbit IgG secondary antibody (Thermo Fisher Scientific, USA). Antibody binding was exposed to an ECL chemiluminescent substrate (Sigma-Aldrich, Singapore) before band images were established using an ImageQuant LAS 500 (GE Healthcare Life Science, Piscataway, NJ, USA).

### Exosome label and uptake

EXs were labeled using an ExoGlow-Membrane EV labeling Kit (System Biosciences, USA), following the manufacturer’s instructions. Briefly, labeling reaction buffer and labeling dye were added into 100 μg of EX and incubated at RT in a dark room for 30 minutes. EXs were loaded into the PD SpinTrap G-25 column (Cytiva Sweden, UK) to remove excess dye and centrifugated at 800 × *g* for two minutes. Labeled EXs were harvested in the first fraction.

EX uptake experiments were performed on human umbilical vein endothelial cells (hUVECs) and human dermal fibroblasts provided by the EV group (Vinmec HiTech Center) and seeded into a 96-well plate before adding the labeled EXs. After four hours of incubation at 37°C, excess media containing labeled EXs and cells were removed and washed with PBS. Then, cells were fixed with PFA 4% for 20 minutes before the nucleus of these cells was stained with DAPI (4′,6-diamidino-2-phenylindole) (Thermo Fisher Scientific). EX uptake was evaluated by using confocal microscopy.

### Growth factor analysis using Luminex assay

Luminex assay using ProcartaPlex^TM^ Multiplex Immunoassays (Human Custom ProcartaPlex 7-Plex Kit) (ThermoFisher, USA) was performed to quantify six growth factors and cytokines including, FGF-2, HGF, VEGF-A, PDGF-BB, IL-6, IL-7, and IL-8) in exosomes. Briefly, capture beads were coated in each well. Then, exosome suspension was added and incubated for two hours before being washed by washing buffer to remove unbound reagents. Details of procedures followed the manufacturer’s instructions. The luminescent signal was monitored using the Luminex^TM^ 100/200^TM^ system with xPONENT 3.1 software.

### Growth factor analysis using ELISA assay

The keratinocyte growth factor (KGF) level in different exosomes was measured using ELISA assay (Abcam, UK). Briefly, the exosome solution was added into each well for 2.5 hours; then, unbound reagents were removed and washed with a washing buffer. A biotinylated secondary antibody was added before streptavidin-conjugated Horseradish Peroxidase. The degree of KGF was measured based on the absorbance measurement (SpectraMax M5, Molecular Devices, Silicon Valley, USA) at 450 nm. The protein amount was determined to be equivalent to the standard curve.

### Proliferation assay

Human dermal fibroblasts were seeded into a 96-well plate (2500 cells/well) with EV-depleted media (5% EV-depleted FBS/PLT by centrifuging at 120.000 x g/ 18 hours in DMEM/F12) containing 10 μg/mL EXs from the culture media listed in Table 1. Fibroblasts were incubated at 37°C and 5% CO_2_ for 48 hours to proliferate. Then, the proliferation rate was evaluated using a 3-(4,5-dimethylthiazol-2-yl)-2,5-diphenyl tetrazolium bromide (MTT) assay (Abcam, UK). The cell proliferation rate was assessed based on the absorbance measurement (SpectraMax M5, Molecular Devices, Silicon Valley, USA) at 560 nm.

### Migration assay

Human dermal fibroblasts were cultured in a 24-well plate with a density of 150,000 cells/well for attachment and expansion. After reaching 100% confluency, cell proliferation was inhibited by Mitomycin C (10 μg/mL) for two hours. Then, a scratch was created by a sterile 100 μL tip before cells were treated with EV-depleted media (5% EV-depleted FBS/PLT by centrifuging at 120.000 x g/ 18 hours in DMEM/F12) containing 10 μg/mL EXs as listed in Table 1. The migration of cells to close the wounded area was recorded using an inverted microscope at various time intervals. The wound area was analyzed using ImageJ software (version 1.48) and evaluated for the closure percentage for several time points.

### Angiogenesis assay

The tube formation assay was performed using an *in vitro* Angiogenesis Assay Kit (Abcam, England). Briefly, the extracellular matrix solution was coated in a 96-well plate for one hour at 37°C. Then, hUVECs were seeded at 15,000 cells/well in different media: EBM-2 (endothelial basal media 2) and EBM-2 supplement (Lonza, Switzerland) with 10 μg/mL EXs listed in Table 1. For control well, no extracellular matrix was coated. The tube formation was evaluated at five hours and 10 hours of incubation at 37°C. Total tube length and total branching points were evaluated by ImageJ software.

### Statistical analysis

GraphPad Prism 9 (GraphPad Software, California, USA) with One-Way and Two-Way ANOVA, and Tukey HSD tests were used to evaluate the statistically significant differences between groups. p < 0.05 was considered as statistical significance. All data were shown as means ± SD.

## Results

### Characterization of UCMSCs under different culture media

**Fig 1:**
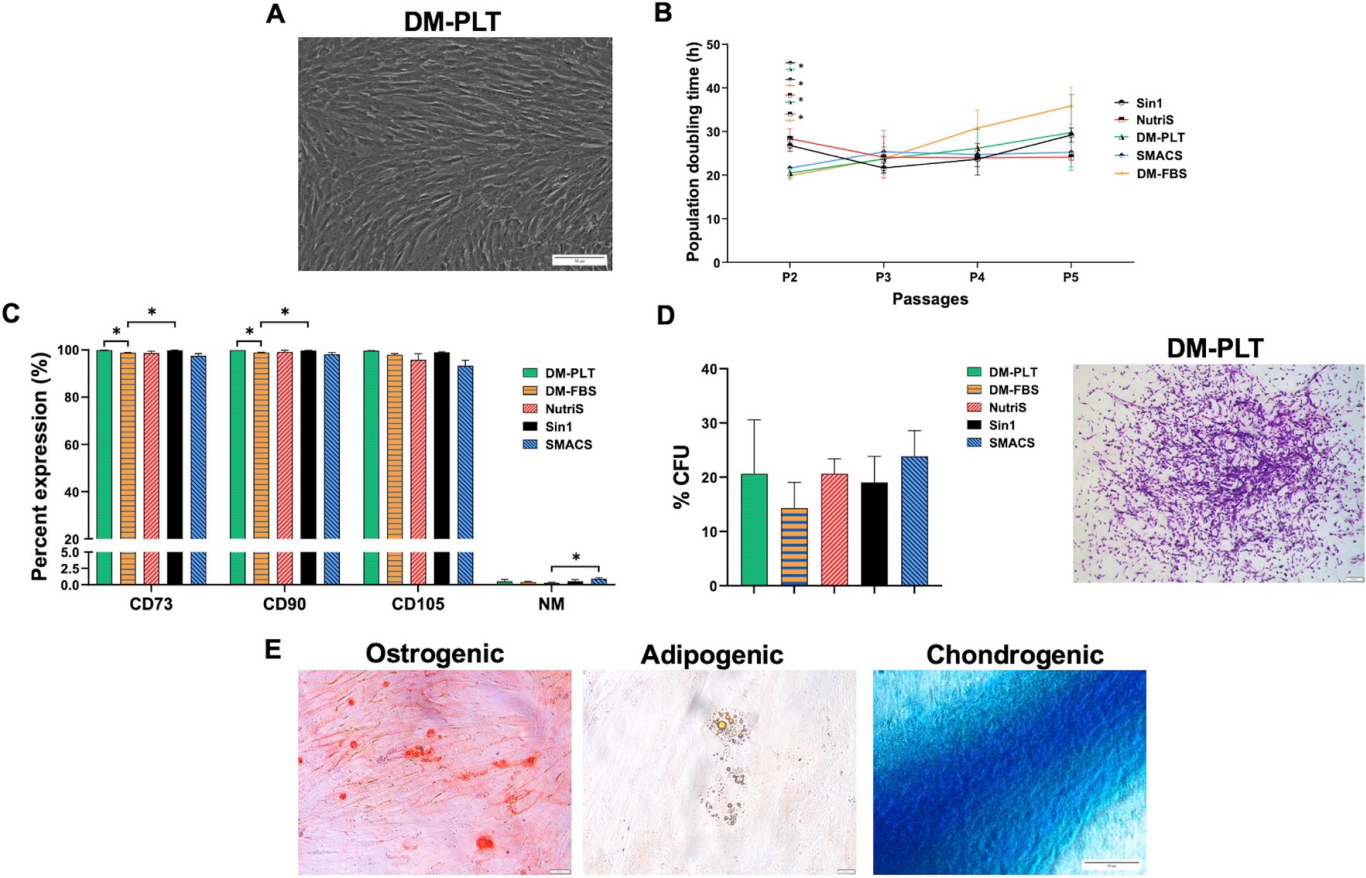
Characteristic of UCMSCs cultured in five different media. (A) A representative morphology of UCMSCs cultured in DM-PLT medium at P5. Magnification 10X. Scale bar 50 μm. (B) A comparative population doubling time of three UCMSC lines cultured in 10% FBS supplemented (DM-FBS), a 5% PLT supplemented (DM-PLT), and three serum-free media (STEMin1, NutriStem, and StemMACS) (n = 3). (C) Expression of MSC surface markers (n = 3) and (D) colony-forming potential of UCMSCs cultured in five different culture media (n = 3). A representative image from DM-PLT culture medium independent experiments is shown. Magnification 4X. Scale bar 200 μm. (E) A representative trilineage differentiation of UCMSCs cultured in DM-PLT medium. Magnification 10X. Scale bar 50 μm. Sin1, NutriS, DM-PLT, SMACS, DM-FBS are UCMSCs cultured in STEMin1, NutriStem, DMEM/F12 medium supplemented with 5% PLT, StemMACS and DMEM/F12 medium supplemented with 10% FBS, respectively. P: Passage, CFU: Colonies forming unit.

The cell culture medium has been shown to largely influence the proliferation and functional properties of MSCs. We conducted an in-depth comparative analysis of the morphology, expansion rate, surface markers, and differentiation potency of primary UCMSCs cultured up to passage 5 (P5) in three categories of culture media, including a 10% FBS supplemented (DM-FBS), a 5% PLT supplemented (DM-PLT) and three serum-free media (STEMin1, NutriStem, and StemMACS) (Table 1). We observed that UCMSCs cultured from passage 1 to passage 5 in these culture media displayed the typical spindle-shaped morphology and similar size (Fig 1A and S1 Fig).

To evaluate the growth rate of UCMSCs cultured in different culture media, we analyzed the population doubling time (PDT) of UCMSCs from P2 to P5. Cells generally showed similar doubling times in all five culture conditions (Fig 1B). However, UCMSCs cultured in STEMin1 at P2 exhibited a longer PDT compared to cells cultured in DM-PLT and StemMACS (p < 0.05). Additionally, the PDT of UCMSCs cultured in DM-PLT was lower than those in NutriStem (p < 0.05). Although the growth rate of UCMSCs cultured in FBS supplemented medium was the highest at P2, the later passages demonstrated a lower proliferation rate than others (Fig 1B).

In addition, UCMSCs cultured in all five media at P3 expressed robust MSC markers, including CD90, CD105, and CD73 (> 95%), except for CD105, which was 93% in UCMSCs maintained in StemMACS medium. Additionally, minimal expression (< 2%) of antigens recognized by the negative marker cocktail, including CD45, CD34, CD11b, CD19, and HLA-DR, was detected (Fig 1C, S2 Fig). To assess the maintenance of stemness properties, we analyzed the colony-forming efficiency of UCMSCs using CFU-F assays. UCMSCs cultured in all five media at passage 3 formed comparable frequencies of CFUs (Fig 1D and S3 Fig). Furthermore, we investigated the multipotency of UCMSC cultures by differentiating them into adipogenic, chondrogenic, and osteogenic lineages. The cells in all five culture medium conditions exhibited the capacity to differentiate into the three cell lineages as indicated by positive staining of Oil Red O, Alizarin Red S, and Alcian Blue (Fig 1E and S4 Fig).

Together, we observed that UCMSCs cultured in three categories of culture medium displayed comparable proliferation, morphology, expression of MSC surface markers, and maintenance of stemness.

### UCMSC-derived exosome characterization

**Fig 2:**
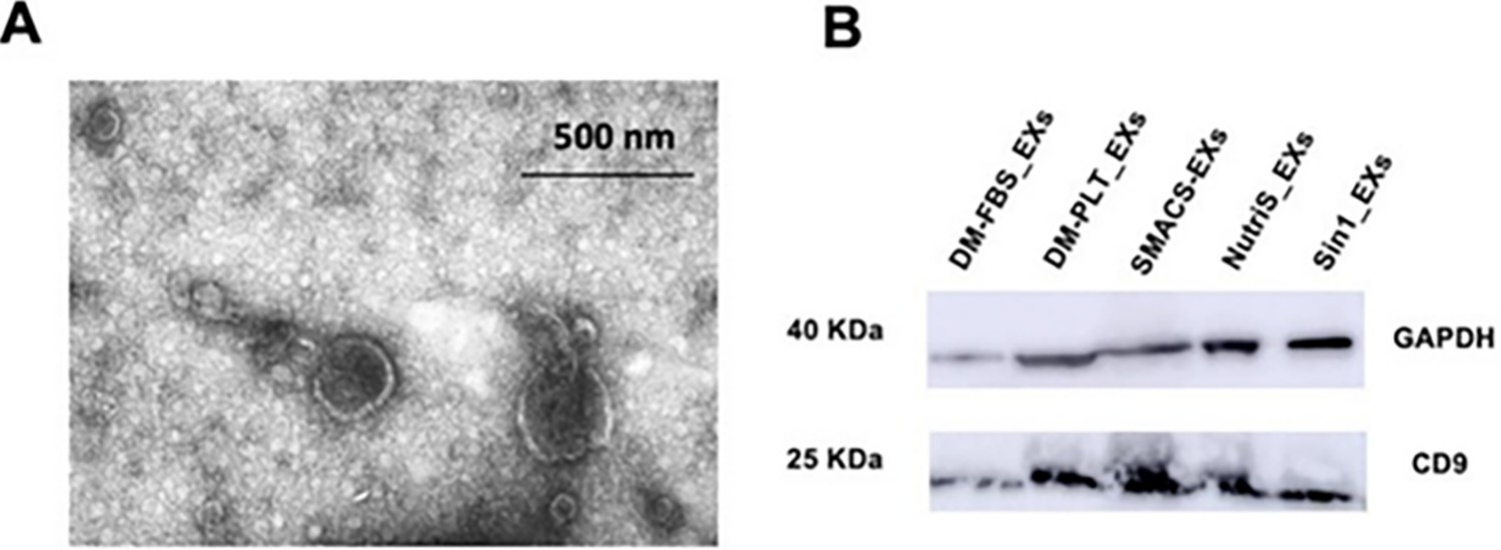
Exosomes derived from UCMSCs displayed typical exosome morphology and surface marker. (A) Representative morphology of EXs derived from UCMSCs cultured in DM-PLT medium observed under transmission electron microscopy. (B) Internal control GAPDH and CD9 were detected in all EX samples with 15 μg total exosomal protein loaded into each lane. DM-FBS_EXs, DM-PLT_EXs, SMACS_EXs, NutriS_EXs, Sin1_EXs are EXs derived from UCMSCs cultured in DMEM/12 + 10% FBS, DMEM/F12 + 5% PLT, StemMACS, NutriStem, and STEMin1, respectively.

We next analyzed the morphology of EXs derived from UCMSCs cultured in five different media (Table 1) by transmission electron microscopy (TEM). The vesicles demonstrated the typical cup-shaped morphology ranging from 40 to 250 nm (Fig 2A). Total exosomal proteins were measured (S5 Fig), before being loaded into each lane in 4–12% NuPAGE gels. The isolated EXs exhibited the typical EX surface protein marker CD9 and the internal reference protein of GAPDH (Fig 2B, S6 Fig), indicating that UCMSC-derived EXs were successfully extracted.

### The differential levels of growth factors in exosomes derived from different media

**Fig 3:**
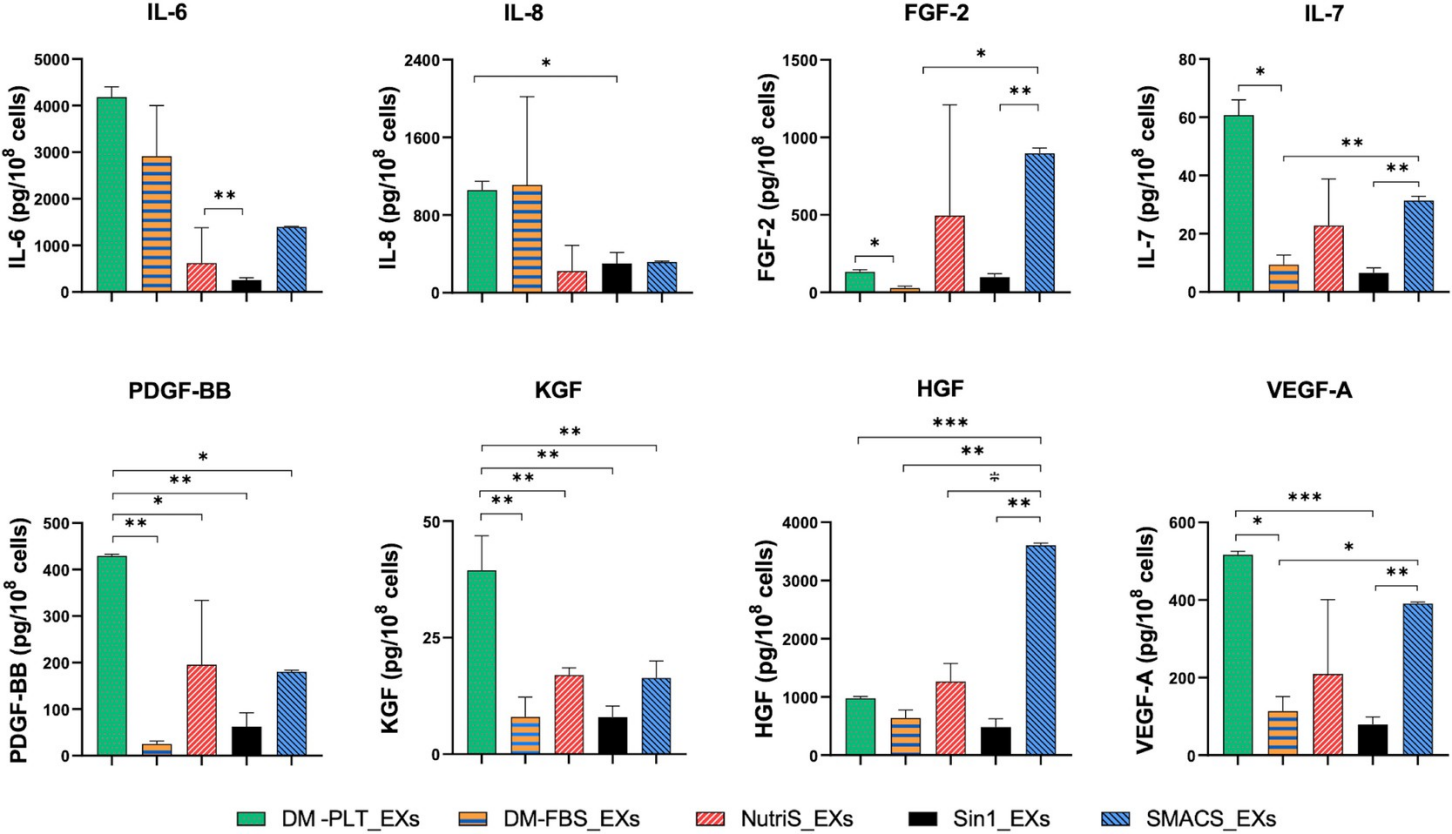
Quantity of growth factors and cytokines in exosomes purified from 10^8^ UCMSCs cultured in different media (n = 3). DM-FBS_EXs, DM-PLT_EXs, SMACS_EXs, NutriS_EXs, Sin1_EXs are EXs from UCMSCs cultured in DMEM/12 + 10% FBS, DMEM/F12 + 5% PLT, StemMACS, NutriStem, and STEMin1, respectively. Statistical significance was determined by ANOVA and post-hoc Tukey HSD tests and is indicated by: * where p < 0.05; ** where p < 0.01; *** where p < 0.001; **** where p < 0.0001.

We next compare the secretome of UCMSC-derived EXs isolated from five different culture media, focusing on a panel of growth factors and cytokines, including FGF-2, KGF, HGF, VEGF-A, IL-6, IL-7, IL-8, and PDGF-BB. We separated EXs from five different culture media and normalized the EX levels with 10^8^ secreting cells to evaluate growth factors, and cytokines encapsulated in EXs using the Luminex platform and ELISA. We revealed that EXs from PLT-supplemented DMEM/F12 exhibited higher concentrations of IL-6, IL-7, IL-8, VEGF-A, PDGF-BB, and KGF than EXs from other media (Fig 3). Besides, HGF and FGF-2 were found in greater levels in EXs from SMACS medium than in EXs from other tested media (Fig 3).

Together, we found that PLT-supplemented medium supported a higher content of various cytokines and growth factors in UCMSC-derived EXs than conventional FBS-supplemented and serum-free media.

### UCMSC-derived exosomes from different culture conditions stimulate fibroblast proliferation

**Fig 4:**
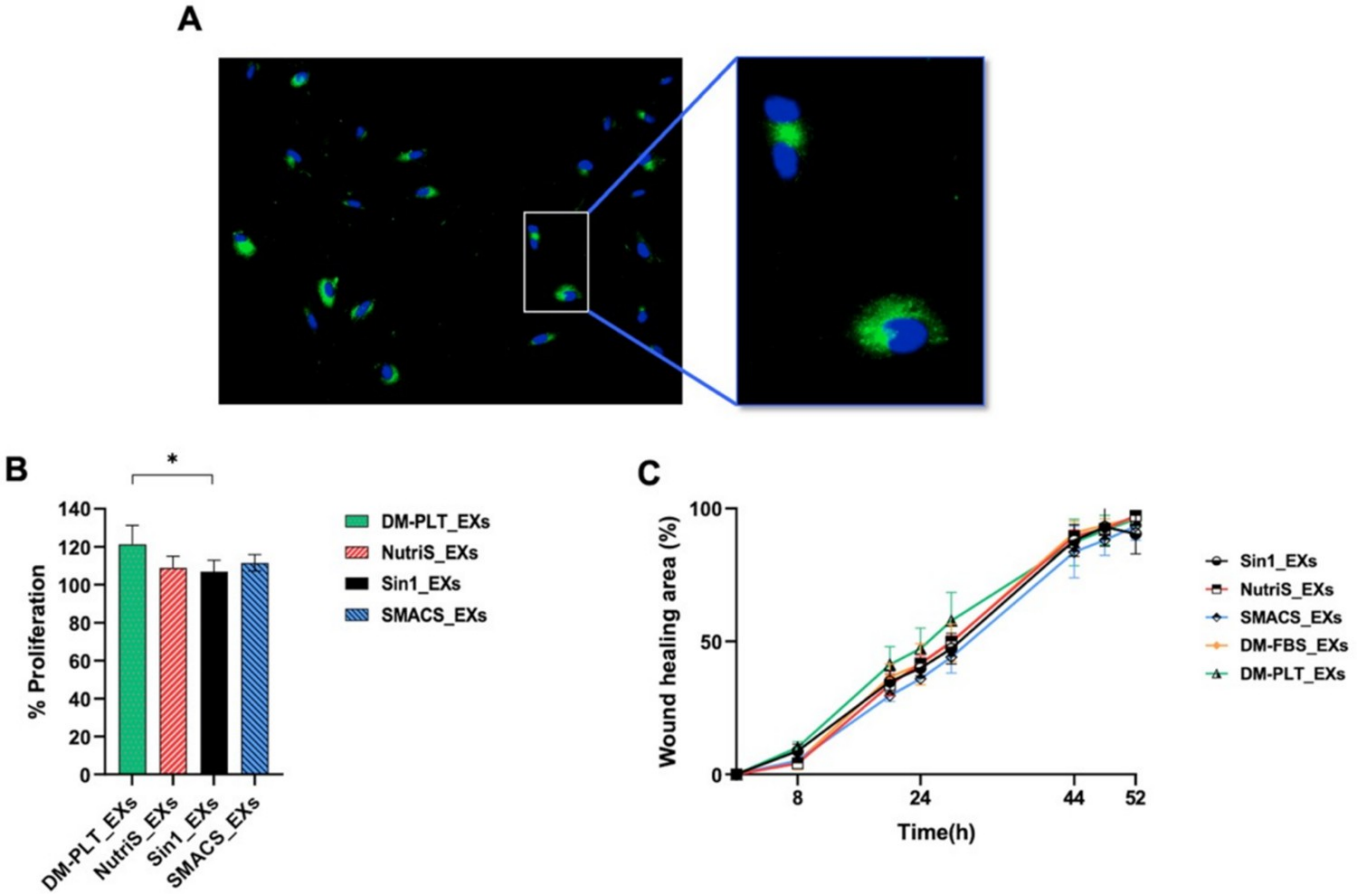
Effects of EXs on fibroblast proliferation and migration (n = 3). (A) Confocal images of fibroblasts incubated for two hours with ExoGlow (green) labeled UCMSC-derived EXs. DAPI was used to stain nuclei (B) Fibroblast proliferation rates induced by EXs from different media. The percentage of cell proliferation was normalized to medium supplement with DM-FBS_EXs. (C) EXs-mediated fibroblast migration as determined by a scratch assay. DM-FBS_EXs, DM-PLT_EXs, SMACS_EXs, NutriS_EXs, Sin1_EXs are EXs from UCMSCs cultured in DMEM/12 + 10% FBS, DMEM/F12 + 5% PLT, StemMACS, NutriStem, and STEMin1, respectively. Statistical significance was determined by ANOVA and post-hoc Tukey HSD tests, two-way ANOVA, and is indicated by: * where p < 0.05; ** where p < 0.01; *** where p < 0.001; **** where p < 0.0001.

Next, we investigated how different exosomal cytokine profiles of EXs from different culture media impact EX-mediated fibroblast proliferation and migration. UCMSC-derived EXs were labeled with ExoGlow-membrane green dye and introduced into the fibroblast culture for 2 hours to assess cell proliferation. We observed an apparent internalization of EXs by fibroblasts (Fig 4A). Furthermore, compared with the DM-FBS_EXs, EXs derived from four other media demonstrated an enhanced capacity to promote fibroblast proliferation. Notably, DM-PLT_EXs exhibited the most remarkable ability to facilitate fibroblast proliferation (Fig 4B).

In addition to fibroblast proliferation, a scratch assay was employed to examine the influence of different EXs on fibroblast migration (Fig 4C and S7 Fig). The results indicated that EXs from PLT-supplemented DMEM/F12 tended to enhance fibroblast migration between 20 to 28 hours compared to other conditions. However, there was no significant difference in the rates of EX-mediated fibroblast migration at any treatment time point among EXs isolated from different UCMSC culture media (Fig 4C).

### UCMSC-exosomes from NutriStem media have a higher angiogenesis effect

**Fig 5:**
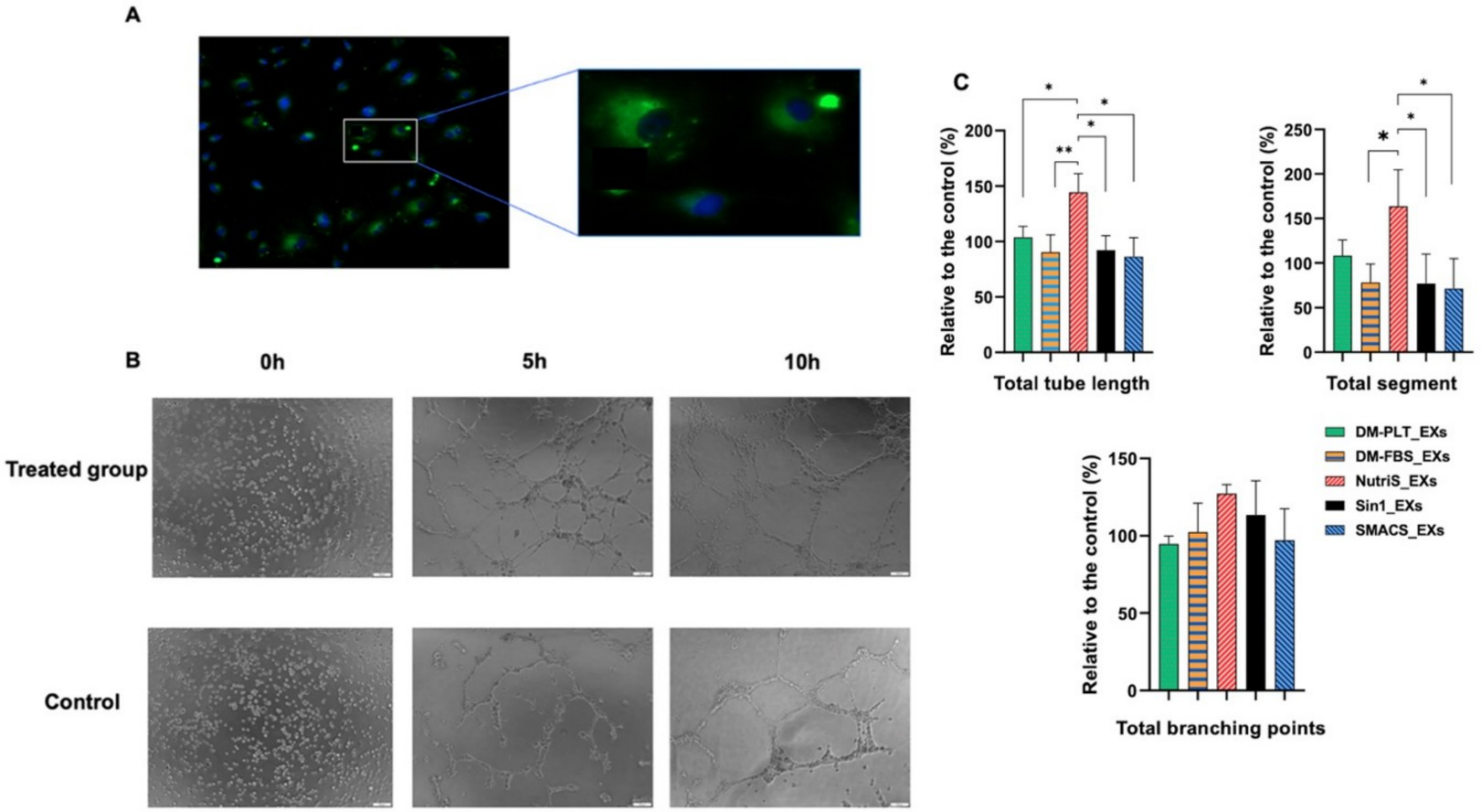
Influence of EX treatment on the tube formation (n = 3). (A) Uptake of ExoGlow-labeled EXs (green) in endothelial cells after two hours. The nucleus was stained with DAPI. (B) The representative tube structures of hUVECs under an inverted microscope. (C) Quantitative analysis of hUVEC tube formation normalized to the control group. DM-FBS_EXs, DM-PLT_EXs, SMACS_EXs, NutriS_EXs, Sin1_EXs are EXs from UCMSCs cultured in DMEM/12 + 10% FBS, DMEM/F12 + 5% PLT, StemMACS, NutriStem, and STEMin1, respectively. Statistical significance was determined by ANOVA and post-hoc Tukey HSD tests and is indicated by: * where p < 0.05; ** where p < 0.01; *** where p < 0.001; **** where p < 0.0001.

Since angiogenesis is an essential step in wound healing, we evaluated the influence of EXs from different culture media on the invitro angiogenic process using human umbilical vein endothelial cells (HUVECs). Similar to fibroblasts, the endothelial cells uptake ExoGlow-labeled EXs (Fig 5A).

We then performed an *in vitro* angiogenesis assay to examine the impact of UCMSC-derived EXs from various culture media on tube formation. The results revealed that DM-PLT_EXs and NutriS_EXs enhanced angiogenesis in hUVECs as evidenced in terms of total tube length and total segment when compared to the control of hUVECs cultured in EBM-2 (Fig 5C). However, there was no significant difference between the treated and control groups regarding the formation of branching points. In addition, the NutriS_EXs demonstrated the highest capacity in promoting tube length and segment formation compared to other treated groups (Fig 5C).

## Discussion

Exosomes (EXs) derived from UCMSCs contain essential factors for various wound healing-associated biological processes. For example, UCMSC-derived EXs, containing VEGF and HIF-1, have demonstrated efficacy in enhancing angiogenesis in rat models [23] and promoting fibroblast proliferation [22, 24]. However, the quantification and profile of secreted factors in EXs can vary depending on the physiological properties of secreting cells and cell culture medium. Remarkably, the type of culture medium can significantly impact the proteins packed into exosomes. For example, ADMSC cultivated in a medium without FBS were found to secrete proteins that could be packed into exosomes at a twofold higher rate than those cultivated with FBS [25]. Therefore, in this study, we conducted a comprehensive analysis to explore the influence of culture media- specifically, FBS-supplemented DMEM/F12, PLT-supplemented DMEM/F12, StemMACs, NutriStem, and STEMin1- on the characteristics of UCMSC-derived EXs and their wound-healing ability. We demonstrated that all five types of culture media were suitable for UCMSC expansion. Importantly, EXs derived from these media exhibited a cup-shaped morphology and expressed CD9 protein marker. Additionally, we detected the presence of growth factors and cytokines packed into these EXs, with DM-PLT_EXs displaying particularly abundant expressions of these cytokines. Consequently, EXs manufactured using the PLT-supplemented medium demonstrated enhanced capability in supporting fibroblast proliferation and blood vessel formation.

We initiated our study by characterizing the proliferation, morphology, MSC surface marker expression, and stemness of three UCMSC lines cultured in five distinct media. Across varied culture media, all the UCMSC lines exhibited typical MSC morphology and adhesion properties. Regarding the proliferation rates, UCMSCs demonstrated robust growth in all tested media, with the population doubling times (PDTs) ranging from 24.12 to 27.55 hours during extended maintenance. Notably, these proliferation rates surpass the range reported in several previous studies, where PDTs of UCMSCs varied widely from 20 to 40 hours in several initial passages, [26–28] possibly due to the different medium used in previous studies. In addition, previous studies suggested that xeno- and serum-free culture media could facilitate a faster expansion of UCMSCs compared to those cultured in FBS-supplemented media [29]. While our data supported this phenomenon from P3 to P5, no significant difference was observed from the other groups.

The assessment of surface markers serves as another critical criterion to determine the quality of MSCs during expansion. We observed that the expression of positive markers, including CD73, CD90, and CD105, exceeded 95%, while negative marker expression remained lower than 2% across all cell cultures. Besides, these UCMSCs demonstrated the fibroblast colony-forming unit (CFU-F) and tri-lineage differentiation capacity. While previous studies showed CFU-formation by UCMSCs and bone marrow-derived MSCs maintained with StemMACS [30], our results represent the first report of CFU-formation by UCMSCs cultured in DMEM/F12 supplemented with 5% PLT and STEMin1. Notably, the findings on the expansion and characteristics of UCMSCs suggest that the three xeno-free media, including NutriStem, STEMin1, and DMEM/F12 supplemented with 5% PLT, are suitable for UCMSCs expansion, both for research and potential clinical applications.

In addition, we revealed that the EXs secreted by UCMSC cultured in all five tested media exhibited the typical cup-shape morphology ranging from 40 to 250 nm, and via TEM observation of the EX-images indicated EX purification. Moreover, EX expressed surface marker CD9, as defined in the ISEV guideline [31]. This finding aligns with other reports characterizing EXs from StemMACS and FBS-supplemented DMEM/F12 medium [8, 10, 22, 32]. Consequently, the data support the reliability and suitability of extracted EXs for further functional analysis.

Previously, it has been demonstrated that the biological functions of MSC-derived EVs are mediated by the presence of their cytokines and growth factors [22, 33–35]. In this study, we identified eight factors, including FGF-2, KGF, HGF, VEGF-A, IL-6, IL-7, IL-8, and PDGF-B, exhibiting higher expression levels in EXs from PLT supplemented medium. These factors have been demonstrated to play essential roles in wound healing-associated biological processes [36]. For instance, HGF can accelerate the proliferation of epithelial and endothelial cells [37] and facilitate β1-integrin/ILK-mediated reepithelialization in epidermal cells [38]. IL-6 possesses pleiotropic activity, contributing to both inflammation and proliferation phases in wound healing. It is produced during the initial inflammation stage, promoting cytotoxic T cells and B cell differentiation to eliminate bacterial infection [39]. Beyond its inflammatory effects, IL-6 plays a pivotal role in orchestrating the polarization of M1 macrophages, predominant in the inflammatory phase, to the M2 phenotype in the proliferation phase [40].

Furthermore, we detected substantial levels of other well-known wound healing-associated factors, such as FGF, VEGF, and PDGF, within UCMSC-derived EXs from various media. These growth factors could promote cell proliferation and migration, as well as regulate the blood-vascular system’s development [41, 42]. Additionally, we detected KGF, a growth factor belonging to the FGF family, which plays a central role in numerous biological processes and signaling pathways involved in the development and regeneration of various tissues [43].

In addition to the high-expression growth factors and cytokine expressed in EXs, we identified two other cytokines, IL-7 and IL-8. IL-7 is essential for T cell development and maintenance of mature T cell balance [44]. It could promote T cell proliferation, thereby improving the clearance of viral and bacterial infections. Notably, IL-7 administration has shown promise in addressing various conditions, including sepsis and HIV or hepatitis C and B infections [45–47]. On the other hand, IL-8, a pro-inflammatory cytokine, primarily functions by attracting neutrophils to inflammatory sites [48]. This cytokine also significantly fosters the growth and differentiation of monocytes and macrophages [48]. The detection of five growth factors, FGF-2, KGF, HGF, PDGF-BB, and VEGF-A, and three cytokines, IL-6, IL-7, and IL-8, in UCMSC-derived EXs collected from various culture media suggests that these EXs might be used to facilitate multiple phases of the wound healing process, spanning from inflammatory to proliferative, and even to remodeling phases. However, it is essential to note that the mechanisms underlying the actions of these analyzed factors in wound healing have yet to be examined.

During the healing process, neighboring cells must proliferate rapidly and migrate toward the wound sites to exhibit their healing functions. Among proposed assays, MTT is widely used to measure cell viability over a prolonged period, and the scratch assay stands out as one of the earliest, most cost-effective, and most reliable methods for investigating directional cell migration in 2D cultures [49–51]. Therefore, in this study, we conducted scratch-based migration and MTT-based proliferation assays to evaluate the effect of UCMSC-derived EXs on the *in vitro* cutaneous wound healing process. We observed the penetration of EX into fibroblast and HUVECs; however, we did not calculate the percentage of cells that uptake the EXs and how many EXs were internalized into the cells. In addition, EXs from both defined and undefined culture medium groups enhanced fibroblast proliferation compared to the conventional culture medium, with DM-PLT_EXs showing particularly pronounced effects (Fig 4B). These findings align with the observed higher growth factor levels in DM-FBS_EXs compared to EXs from other media (Fig 3). Furthermore, EXs produced in PLT supplemented medium exhibited superior capabilities in promoting fibroblast proliferation compared to Sin1_EXs (Fig 4B), attributed to elevated levels of KGF, IL8, and PDGF-BB (Fig 3). On the other hand, UCMSC-derived EXs isolated from five tested media did not exhibit significant differences in supporting the dermal fibroblast migration (Fig 4C). To our knowledge, this is the sole study assessing the influence of culture medium on the functionality of EXs in the context of the fibroblast migration process.

Angiogenesis plays a vital role in wound healing by facilitating the supply of nutrients and oxygen to newly formed tissues [52]. Endothelial cells primarily drive this process, and VEGF-A emerges as a critical regulator [53]. The newly sprouting blood vessels resulting from angiogenesis provide increased nutrients and oxygen to cells involved in the wound-healing process, such as fibroblasts, thereby promoting their proliferation and migration. Therefore, beyond directly promoting fibroblast migration and proliferation, EXs could potentially have a synergistic effect in fostering fibroblast functions in wound healing by augmenting angiogenesis. Consequently, in this study, we conducted angiogenesis assays to elucidate the impact of EXs on wound healing. Among investigated EXs, NutriS_EXs demonstrated the most potent promotion of tube length and segment formation compared to the others (Fig 5C). Besides, DM-PLT_EXs also enhanced tube length formation compared to the control (Fig 5C). Surprisingly, the observed effect of EXs on angiogenesis does not align consistently with the levels of VEGF-A in EXs from different media. Apart from VEGF-A, other exosomal factors may play a role in EX-mediated angiogenesis, as reported in previous studies [54]. Hence, further studies are necessary to investigate the impact of exosomal factors on angiogenesis comprehensively.

In conclusion, our study represents the investigation into the impact of various xeno-free culture media on UCMSC expansion and the roles of EXs derived from these media in the wound healing process. All culture media exhibited comparable efficacy in maintaining UCMSC characteristics, including stemness, the typical spindle-shaped morphology, surface markers, high proliferation rates, CFU formation, and tri-linage differentiation. However, UCMSC-derived EXs from PLT-supplemented DMEM/F12 demonstrated enriched levels of growth factors such as KGF, VEGF-A, IL-6, IL-7, and PDGF-BB. Notably, these DM-PLT_EXs demonstrated an enhanced capacity to promote fibroblast proliferation compared with DM-FBS_EXs and Sin1_EXs. In addition, NutriS_EXs proved to be the most effective in enhancing the angiogenesis process, alongside EXs from PLT-supplemented DMEM/F12. Our findings suggest that the PLT-supplemented DMEM/F12 medium may be the most suitable choice for manufacturing MSC-derived EXs for clinical applications. However, our limitation is due to the nature of exosomes and technical issues as the large variation in exosomal proteins detected in different samples (S5 Fig). Therefore, replicating more biological samples in the future is required to address this issue. More importantly, the components of these commercially available GMP culture media are undetermined; this presents a challenge in assessing how the specific components influence the growth of primary MSCs, the secretion of EXs, and the concentration of growth factors within these EXs. Consequently, it becomes difficult to evaluate the impact of individual components on these processes thoroughly.

## Supporting information

S1 Fig Morphology of UCMSCs cultured in different media from P1 to P5. Sin1, NutriS, DM-PLT, SMACS, DM-FBS are UCMSCs cultured in STEMin1, NutriStem, DMEM/F12 medium supplemented with 5% PLT, StemMACS and DMEM/F12 medium supplemented with 10% FBS, respectively.
(TIF)

S2 Fig A representative marker analysis of UCMSCs cultured in DM-PLT medium at P3. (TIF)

S3 Fig Morphology of CFU formed from cells cultured in different media. Sin1, NutriS, DM-PLT, SMACS, DM-FBS are UCMSCs cultured in STEMin1, NutriStem, DMEM/F12 medium supplemented with 5% PLT, StemMACS and DMEM/F12 medium supplemented with 10% FBS, respectively.
(TIF)

S4 Fig Trilineage differentiation of isolated UCB-MSCs. MSCs were cultured in osteogenic, adipogenic, and chondrogenic induction media and stained with corresponding staining dyes. Sin1, NutriS, DM-PLT, SMACS, DM-FBS are UCMSCs cultured in STEMin1, NutriStem, DMEM/F12 medium supplemented with 5% PLT, StemMACS and DMEM/F12 medium supplemented with 10% FBS, respectively.
(TIF)

S5 Fig Exosomal protein concentration from different types of culture media normalized with 10^8^ cells. DM-FBS_EXs, DM-PLT_EXs, SMACS_EXs, NutriS_EXs, Sin1_EXs are EXs from UCMSCs cultured in DMEM/12 + 10% FBS, DMEM/F12 + 5% PLT, StemMACS, NutriStem, and STEMin1, respectively.
(TIF)

S6 Fig The expression of CD9 and GAPDH from different types of UCMSC_derived EXs. DM-FBS_EXs, DM-PLT_EXs, SMACS_EXs, NutriS_EXs, Sin1_EXs are EXs from UCMSCs cultured in DMEM/12 + 10% FBS, DMEM/F12 + 5% PLT, StemMACS, NutriStem, and STEMin1, respectively.
(TIF)

S7 Fig EXs-mediated fibroblast migration as determined by a scratch assay at 0h, 20h, 28h, 52h. DM-FBS_EXs, DM-PLT_EXs, SMACS_EXs, NutriS_EXs, Sin1_EXs are EXs from UCMSCs cultured in DMEM/12 + 10% FBS, DMEM/F12 + 5% PLT, StemMACS, NutriStem, and STEMin1, respectively.
(TIF)

## References

[1] Than UTT, Guanzon D, Leavesley D, Parker T. Association of Extracellular Membrane Vesicles with Cutaneous Wound Healing. Int J Mol Sci. 2017;18: 956. doi: 10.3390/ijms18050956 28468315 PMC5454869

[2] Frykberg RG, Banks J. Challenges in the Treatment of Chronic Wounds. Adv Wound Care. 2015;4: 560–582. doi: 10.1089/wound.2015.0635 26339534 PMC4528992

[3] Nagamura-Inoue T, He H. Umbilical cord-derived mesenchymal stem cells: Their advantages and potential clinical utility. World J Stem Cells. 2014;6: 195–202. doi: 10.4252/wjsc.v6.i2.195 24772246 PMC3999777

[4] Lee CH, Moioli EK, Mao JJ. Fibroblastic differentiation of human mesenchymal stem cells using connective tissue growth factor. Conf Proc Annu Int Conf IEEE Eng Med Biol Soc IEEE Eng Med Biol Soc Annu Conf. 2006;2006: 775–778. doi: 10.1109/IEMBS.2006.259866 PMC4035038 17946857

[5] De Ugarte DA, Morizono K, Elbarbary A, Alfonso Z, Zuk PA, Zhu M, et al. Comparison of multi-lineage cells from human adipose tissue and bone marrow. Cells Tissues Organs. 2003;174: 101–109. doi: 10.1159/000071150 12835573

[6] Seo K, Sohn S, Bhang D, Nam M, Lee H, Youn H. Therapeutic effects of hepatocyte growth factor‐overexpressing human umbilical cord blood‐derived mesenchymal stem cells on liver fibrosis in rats. Cell Biol Int. 2014;38: 106–116. doi: 10.1002/cbin.10186 24115681

[7] J. Braga Osorio Gomes Salgado A, L. Goncalves Reis R, Jorge Carvalho Sousa N, M. Gimble J, J. Salgado A, L. Reis R, et al. Adipose Tissue Derived Stem Cells Secretome: Soluble Factors and Their Roles in Regenerative Medicine. Curr Stem Cell Res Ther. 2010;5: 103–110. doi: 10.2174/157488810791268564 19941460

[8] Nguyen TH, Dao HH, Duong CM, Nguyen X-H, Hoang DH, Do X-H, et al. Cytokine-primed umbilical cord mesenchymal stem cells enhanced therapeutic effects of extracellular vesicles on osteoarthritic chondrocytes. Front Immunol. 2022;13: 1041592. doi: 10.3389/fimmu.2022.1041592 36389838 PMC9647019

[9] Suh JH, Joo HS, Hong EB, Lee HJ, Lee JM. Therapeutic application of exosomes in inflammatory diseases. Int J Mol Sci. 2021;22: 1144. Available: https://www.mdpi.com/1422-0067/22/3/1144 doi: 10.3390/ijms22031144 33498928 PMC7865921

[10] Vu DM, Nguyen V-T, Nguyen TH, Do PTX, Dao HH, Hai DX, et al. Effects of extracellular vesicles secreted by TGFβ-stimulated umbilical cord mesenchymal stem cells on skin fibroblasts by promoting fibroblast migration and ECM protein production. Biomedicines. 2022;10: 1810. Available: https://www.mdpi.com/2227-9059/10/8/1810 36009357 10.3390/biomedicines10081810 PMC9405311

[11] Ren S, Chen J, Duscher D, Liu Y, Guo G, Kang Y, et al. Microvesicles from human adipose stem cells promote wound healing by optimizing cellular functions via AKT and ERK signaling pathways. Stem Cell Res Ther. 2019;10: 47. doi: 10.1186/s13287-019-1152-x 30704535 PMC6357421

[12] He X, Dong Z, Cao Y, Wang H, Liu S, Liao L, et al. MSC-derived exosome promotes M2 polarization and enhances cutaneous wound healing. Stem Cells Int. 2019;2019. Available: https://www.hindawi.com/journals/sci/2019/7132708/ doi: 10.1155/2019/7132708 31582986 PMC6754952

[13] Li X, Liu L, Yang J, Yu Y, Chai J, Wang L, et al. Exosome derived from human umbilical cord mesenchymal stem cell mediates MiR-181c attenuating burn-induced excessive inflammation. EBioMedicine. 2016;8: 72–82. Available: https://www.thelancet.com/article/S2352-3964(16)30173-6/abstract doi: 10.1016/j.ebiom.2016.04.030 27428420 PMC4919539

[14] Beninson LA, Fleshner M. Exosomes in fetal bovine serum dampen primary macrophage IL-1β response to lipopolysaccharide (LPS) challenge. Immunol Lett. 2015;163: 187–192. Available: https://www.sciencedirect.com/science/article/pii/S0165247814002387 25455591 10.1016/j.imlet.2014.10.019

[15] Cimino M, Gonçalves RM, Barrias CC, Martins MCL. Xeno-free strategies for safe human mesenchymal stem/stromal cell expansion: supplements and coatings. Stem Cells Int. 2017;2017. Available: https://www.hindawi.com/journals/sci/2017/6597815/abs/ doi: 10.1155/2017/6597815 29158740 PMC5660800

[16] Heiskanen A, Satomaa T, Tiitinen S, Laitinen A, Mannelin S, Impola U, et al. N-glycolylneuraminic acid xenoantigen contamination of human embryonic and mesenchymal stem cells is substantially reversible. Stem Cells. 2007;25: 197–202. Available: https://academic.oup.com/stmcls/article-abstract/25/1/197/6401676 doi: 10.1634/stemcells.2006-0444 17008421

[17] Komoda H, Okura H, Lee CM, Sougawa N, Iwayama T, Hashikawa T, et al. Reduction of *N* -Glycolylneuraminic Acid Xenoantigen on Human Adipose Tissue-Derived Stromal Cells/Mesenchymal Stem Cells Leads to Safer and More Useful Cell Sources for Various Stem Cell Therapies. Tissue Eng Part A. 2010;16: 1143–1155. doi: 10.1089/ten.tea.2009.0386 19863253

[18] Lepperdinger G, Brunauer R, Jamnig A, Laschober G, Kassem M. Controversial issue: is it safe to employ mesenchymal stem cells in cell-based therapies? Exp Gerontol. 2008;43: 1018–1023. Available: https://www.sciencedirect.com/science/article/pii/S0531556508002003 doi: 10.1016/j.exger.2008.07.004 18694815

[19] Bui HTH, Nguyen LT, Than UTT. Influences of Xeno-Free Media on Mesenchymal Stem Cell Expansion for Clinical Application. Tissue Eng Regen Med. 2021;18: 15–23. doi: 10.1007/s13770-020-00306-z 33150562 PMC7862486

[20] Kandoi S, Patra B, Vidyasekar P, Sivanesan D, K R, Verma RS. Evaluation of platelet lysate as a substitute for FBS in explant and enzymatic isolation methods of human umbilical cord MSCs. Sci Rep. 2018;8: 12439. Available: https://www.nature.com/articles/s41598-018-30772-4 doi: 10.1038/s41598-018-30772-4 30127445 PMC6102222

[21] Li Y, Zhang C, Xiong F, Yu M, Peng F, Shang Y, et al. Comparative study of mesenchymal stem cells from C57BL/10 and mdx mice. BMC Cell Biol. 2008;9: 24. doi: 10.1186/1471-2121-9-24 18489762 PMC2415111

[22] Hoang DH, Nguyen TD, Nguyen H-P, Nguyen X-H, Do PTX, Dang VD, et al. Differential wound healing capacity of mesenchymal stem cell-derived exosomes originated from bone marrow, adipose tissue and umbilical cord under serum-and xeno-free condition. Front Mol Biosci. 2020;7: 119. Available: https://www.frontiersin.org/articles/10.3389/fmolb.2020.00119/full 32671095 10.3389/fmolb.2020.00119 PMC7327117

[23] Zhang Y, Hao Z, Wang P, Xia Y, Wu J, Xia D, et al. Exosomes from human umbilical cord mesenchymal stem cells enhance fracture healing through HIF‐1α‐mediated promotion of angiogenesis in a rat model of stabilized fracture. Cell Prolif. 2019;52: e12570. doi: 10.1111/cpr.12570 30663158 PMC6496165

[24] Kim Y-J, mi Yoo S, Park HH, Lim HJ, Kim Y-L, Lee S, et al. Exosomes derived from human umbilical cord blood mesenchymal stem cells stimulates rejuvenation of human skin. Biochem Biophys Res Commun. 2017;493: 1102–1108. Available: https://www.sciencedirect.com/science/article/pii/S0006291X1731817X doi: 10.1016/j.bbrc.2017.09.056 28919421

[25] Lara ML, Carvalho MG, de Souza FF, Schmith RA, Codognoto VM, De Vita B, et al. Influence of culture conditions on the secretome of mesenchymal stem cells derived from feline adipose tissue: Proteomics approach. Biochimie. 2023;211: 78–86. Available: https://www.sciencedirect.com/science/article/pii/S0300908423000664 doi: 10.1016/j.biochi.2023.03.004 36931338

[26] Hendijani F, Sadeghi-Aliabadi H, Haghjooy Javanmard S. Comparison of human mesenchymal stem cells isolated by explant culture method from entire umbilical cord and Wharton’s jelly matrix. Cell Tissue Bank. 2014;15: 555–565. doi: 10.1007/s10561-014-9425-1 24532125

[27] Lee JY, Kang MH, Jang JE, Lee JE, Yang Y, Choi JY, et al. Comparative analysis of mesenchymal stem cells cultivated in serum free media. Sci Rep. 2022;12: 8620. Available: https://www.nature.com/articles/s41598-022-12467-z doi: 10.1038/s41598-022-12467-z 35597800 PMC9124186

[28] Mirabdollahi M, Haghjooyjavanmard S, Sadeghi-aliabadi H. An anticancer effect of umbilical cord-derived mesenchymal stem cell secretome on the breast cancer cell line. Cell Tissue Bank. 2019;20: 423–434. doi: 10.1007/s10561-019-09781-8 31338647

[29] Julavijitphong S, Wichitwiengrat S, Tirawanchai N, Ruangvutilert P, Vantanasiri C, Phermthai T. A xeno-free culture method that enhances Wharton’s jelly mesenchymal stromal cell culture efficiency over traditional animal serum–supplemented cultures. Cytotherapy. 2014;16: 683–691. Available: https://www.sciencedirect.com/science/article/pii/S1465324913006415 doi: 10.1016/j.jcyt.2013.07.012 24119645

[30] Bhat S, Viswanathan P, Chandanala S, Prasanna SJ, Seetharam RN. Expansion and characterization of bone marrow derived human mesenchymal stromal cells in serum-free conditions. Sci Rep. 2021;11: 3403. Available: https://www.nature.com/articles/s41598-021-83088-1 doi: 10.1038/s41598-021-83088-1 33564114 PMC7873235

[31] Lötvall J, Hill AF, Hochberg F, Buzás EI, Di Vizio D, Gardiner C, et al. Minimal experimental requirements for definition of extracellular vesicles and their functions: a position statement from the International Society for Extracellular Vesicles. J Extracell Vesicles. 2014;3: 26913. doi: 10.3402/jev.v3.26913 25536934 PMC4275645

[32] González‐Cubero E, González‐Fernández ML, Gutiérrez‐Velasco L, Navarro‐Ramírez E, Villar‐Suárez V. Isolation and characterization of exosomes from adipose tissue‐derived mesenchymal stem cells. J Anat. 2021;238: 1203–1217. doi: 10.1111/joa.13365 33372709 PMC8053584

[33] Fitzgerald W, Freeman ML, Lederman MM, Vasilieva E, Romero R, Margolis L. A system of cytokines encapsulated in extracellular vesicles. Sci Rep. 2018;8: 8973. Available: https://www.nature.com/articles/s41598-018-27190-x doi: 10.1038/s41598-018-27190-x 29895824 PMC5997670

[34] Petit I, Levy A, Estrach S, Féral CC, Trentin AG, Dingli F, et al. Fibroblast growth factor-2 bound to specific dermal fibroblast-derived extracellular vesicles is protected from degradation. Sci Rep. 2022;12: 22131. Available: https://www.nature.com/articles/s41598-022-26217-8 doi: 10.1038/s41598-022-26217-8 36550142 PMC9780220

[35] Rodrigues-Junior DM, Tsirigoti C, Lim SK, Heldin C-H, Moustakas A. Extracellular vesicles and transforming growth factor β signaling in cancer. Front Cell Dev Biol. 2022;10: 849938. Available: https://www.frontiersin.org/articles/10.3389/fcell.2022.849938/full 35493080 10.3389/fcell.2022.849938 PMC9043557

[36] Park JW, Hwang SR, Yoon I-S. Advanced growth factor delivery systems in wound management and skin regeneration. Molecules. 2017;22: 1259. Available: https://www.mdpi.com/1420-3049/22/8/1259 doi: 10.3390/molecules22081259 28749427 PMC6152378

[37] Miyagi H, Thomasy SM, Russell P, Murphy CJ. The role of hepatocyte growth factor in corneal wound healing. Exp Eye Res. 2018;166: 49–55. Available: https://www.sciencedirect.com/science/article/pii/S0014483517304876 doi: 10.1016/j.exer.2017.10.006 29024692 PMC5831200

[38] Li J-F, Duan H-F, Wu C-T, Zhang D-J, Deng Y, Yin H-L, et al. HGF accelerates wound healing by promoting the dedifferentiation of epidermal cells through-integrin/ILK pathway. BioMed Res Int. 2013;2013. Available: https://www.hindawi.com/journals/bmri/2013/470418/abs/10.1155/2013/470418 PMC3899705 24490163

[39] Okada M, Kitahara M, Kishimoto S, Matsuda T, Hirano T, Kishimoto T. IL-6/BSF-2 functions as a killer helper factor in the in vitro induction of cytotoxic T cells. J Immunol Baltim Md 1950. 1988;141: 1543–1549. Available: https://journals.aai.org/jimmunol/article-abstract/141/5/1543/20906 3261754

[40] Johnson BZ, Stevenson AW, Prêle CM, Fear MW, Wood FM. The role of IL-6 in skin fibrosis and cutaneous wound healing. Biomedicines. 2020;8: 101. Available: https://www.mdpi.com/2227-9059/8/5/101 doi: 10.3390/biomedicines8050101 32365896 PMC7277690

[41] Farooq M, Khan AW, Kim MS, Choi S. The role of fibroblast growth factor (FGF) signaling in tissue repair and regeneration. Cells. 2021;10: 3242. Available: https://www.mdpi.com/2073-4409/10/11/3242 doi: 10.3390/cells10113242 34831463 PMC8622657

[42] Pierce GF, Mustoe TA, Altrock BW, Deuel TF, Thomason A. Role of platelet‐derived growth factor in wound healing. J Cell Biochem. 1991;45: 319–326. doi: 10.1002/jcb.240450403 2045423

[43] Rubin JS, Bottaro DP, Chedid M, Miki T, Ron D, Cheon H-G, et al. Keratinocyte growth factor. Cell Biol Int. 1995;19: 399–412. Available: https://www.sciencedirect.com/science/article/pii/S1065699585710852 doi: 10.1006/cbir.1995.1085 7640656

[44] Mackall CL, Fry TJ, Gress RE. Harnessing the biology of IL-7 for therapeutic application. Nat Rev Immunol. 2011;11: 330–342. Available: https://www.nature.com/articles/nri2970 doi: 10.1038/nri2970 21508983 PMC7351348

[45] Levy Y, Lacabaratz C, Weiss L, Viard J-P, Goujard C, Lelièvre J-D, et al. Enhanced T cell recovery in HIV-1–infected adults through IL-7 treatment. J Clin Invest. 2009;119: 997–1007. Available: https://www.jci.org/articles/view/38052 doi: 10.1172/JCI38052 19287090 PMC2662568

[46] Pellegrini M, Calzascia T, Toe JG, Preston SP, Lin AE, Elford AR, et al. IL-7 engages multiple mechanisms to overcome chronic viral infection and limit organ pathology. Cell. 2011;144: 601–613. Available: https://www.cell.com/abstract/S0092-8674%2811%2900012–2[br]http://timesofindia.indiatimes.com/life-style/health-fitness/health/Boosting-bodys-immune-system-key-to-HIV-cure/articleshow/7425019.cms doi: 10.1016/j.cell.2011.01.011 21295337

[47] Sereti I, Dunham RM, Spritzler J, Aga E, Proschan MA, Medvik K, et al. IL-7 administration drives T cell–cycle entry and expansion in HIV-1 infection. Blood J Am Soc Hematol. 2009;113: 6304–6314. Available: https://ashpublications.org/blood/article-abstract/113/25/6304/25693 doi: 10.1182/blood-2008-10-186601 19380868 PMC2710926

[48] Corre I, Pineau D, Hermouet S. Interleukin-8: an autocrine/paracrine growth factor for human hematopoietic progenitors acting in synergy with colony stimulating factor-1 to promote monocyte-macrophage growth and differentiation. Exp Hematol. 1999;27: 28–36. doi: 10.1016/s0301-472x(98)00032-0 9923441

[49] Rodriguez LG, Wu X, Guan J-L. Wound-Healing Assay. Cell Migration. New Jersey: Humana Press; 2004. pp. 023–030. doi: 10.1385/1-59259-860-9:023

[50] Wiegand C, Abel M, Hipler U-C, Elsner P. Effect of non-adhering dressings on promotion of fibroblast proliferation and wound healing in vitro. Sci Rep. 2019;9: 4320. doi: 10.1038/s41598-019-40921-y 30867534 PMC6416289

[51] Szwedowicz U, Szewczyk A, Gołąb K, Choromańska A. Evaluation of Wound Healing Activity of Salvianolic Acid B on In Vitro Experimental Model. Int J Mol Sci. 2021;22: 7728. doi: 10.3390/ijms22147728 34299351 PMC8307677

[52] Raina N, Rani R, Gupta M. Chapter 8—Angiogenesis: Aspects in wound healing. In: Chatterjee S, editor. Endothelial Signaling in Vascular Dysfunction and Disease. Academic Press; 2021. pp. 77–90. doi: 10.1016/B978-0-12-816196-8.00010-2

[53] Johnson KE, Wilgus TA. Vascular Endothelial Growth Factor and Angiogenesis in the Regulation of Cutaneous Wound Repair. Adv Wound Care. 2014;3: 647–661. doi: 10.1089/wound.2013.0517 25302139 PMC4183920

[54] Carpentier G, Berndt S, Ferratge S, Rasband W, Cuendet M, Uzan G, et al. Angiogenesis Analyzer for ImageJ—A comparative morphometric analysis of “Endothelial Tube Formation Assay” and “Fibrin Bead Assay.” Sci Rep. 2020;10: 11568. doi: 10.1038/s41598-020-67289-8 32665552 PMC7360583

