# Supplementary figures and images for: Manufacturing exosomes for wound healing: Comparative analysis of culture media
Source: PLoS One. 2024 Nov 14;19(11):e0313697. doi: 10.1371/journal.pone.0313697 (PMC11563385; doi:10.1371/journal.pone.0313697)

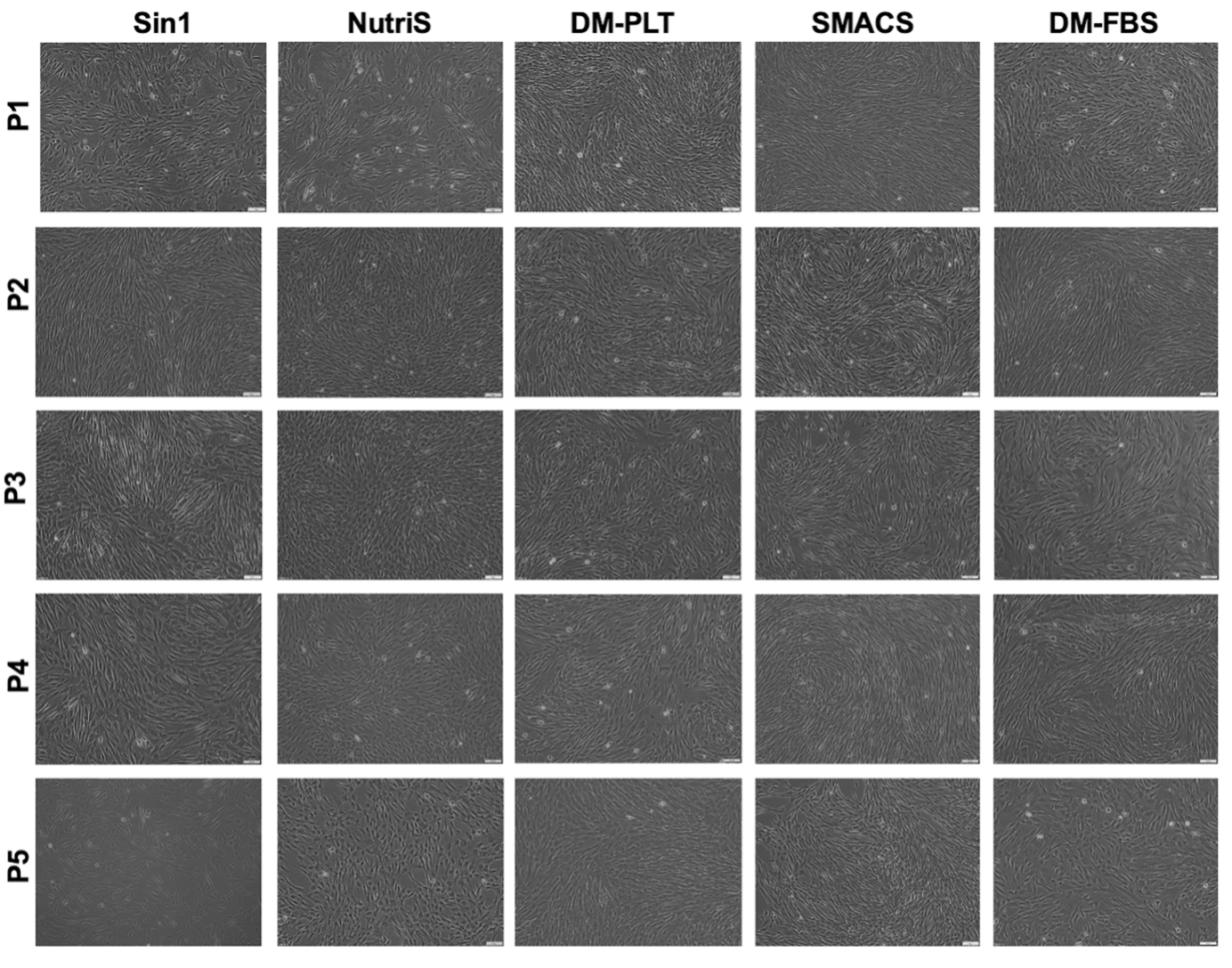

Supplement: S1 Fig — Sin1, NutriS, DM-PLT, SMACS, DM-FBS are UCMSCs cultured in STEMin1, NutriStem, DMEM/F12 medium supplemented with 5% PLT, StemMACS and DMEM/F12 medium supplemented with 10% FBS, respectively. (TIF) [file pone.0313697.s001.tif]

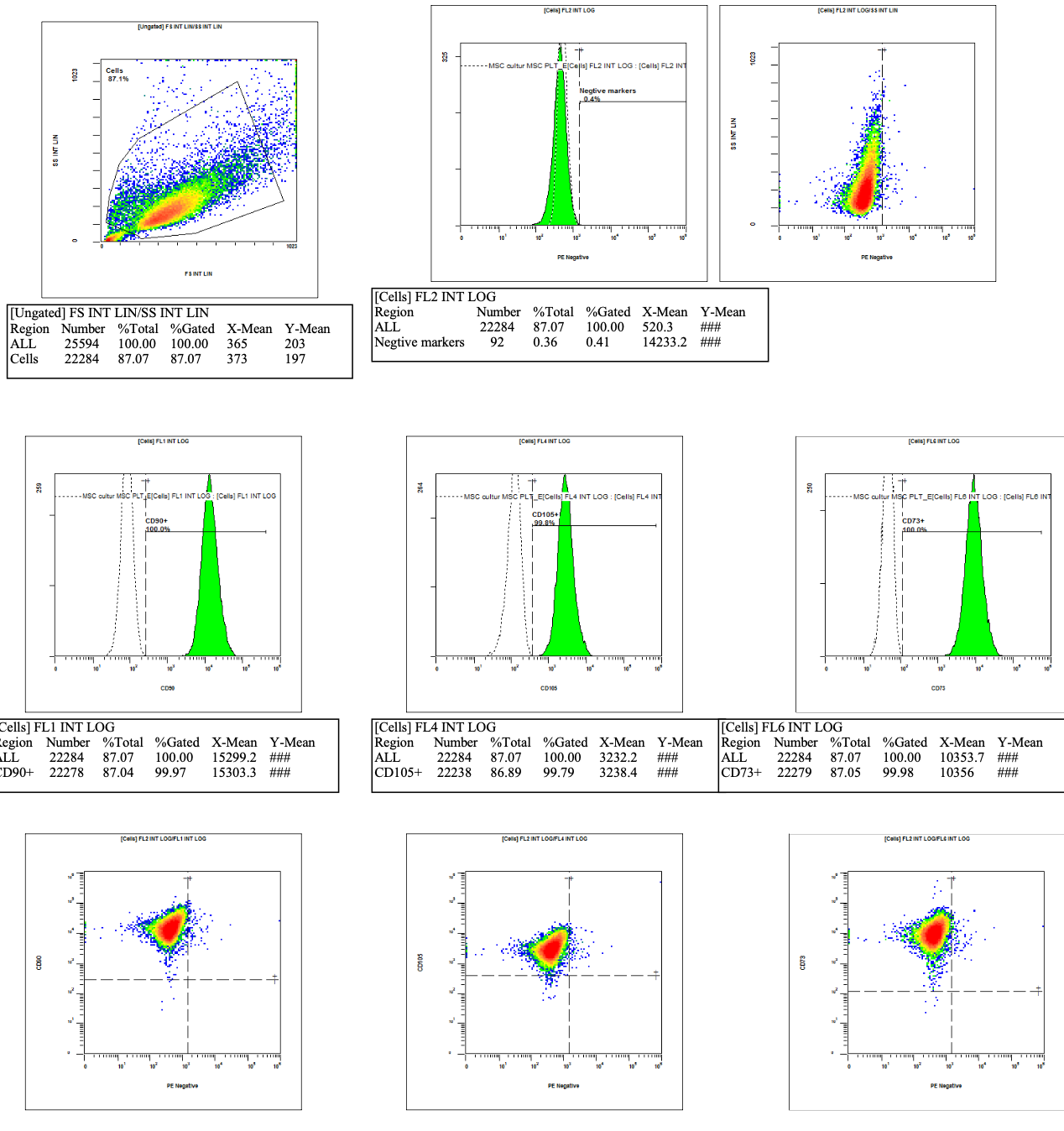

Supplement: S2 Fig — (TIF) [file pone.0313697.s002.tif]

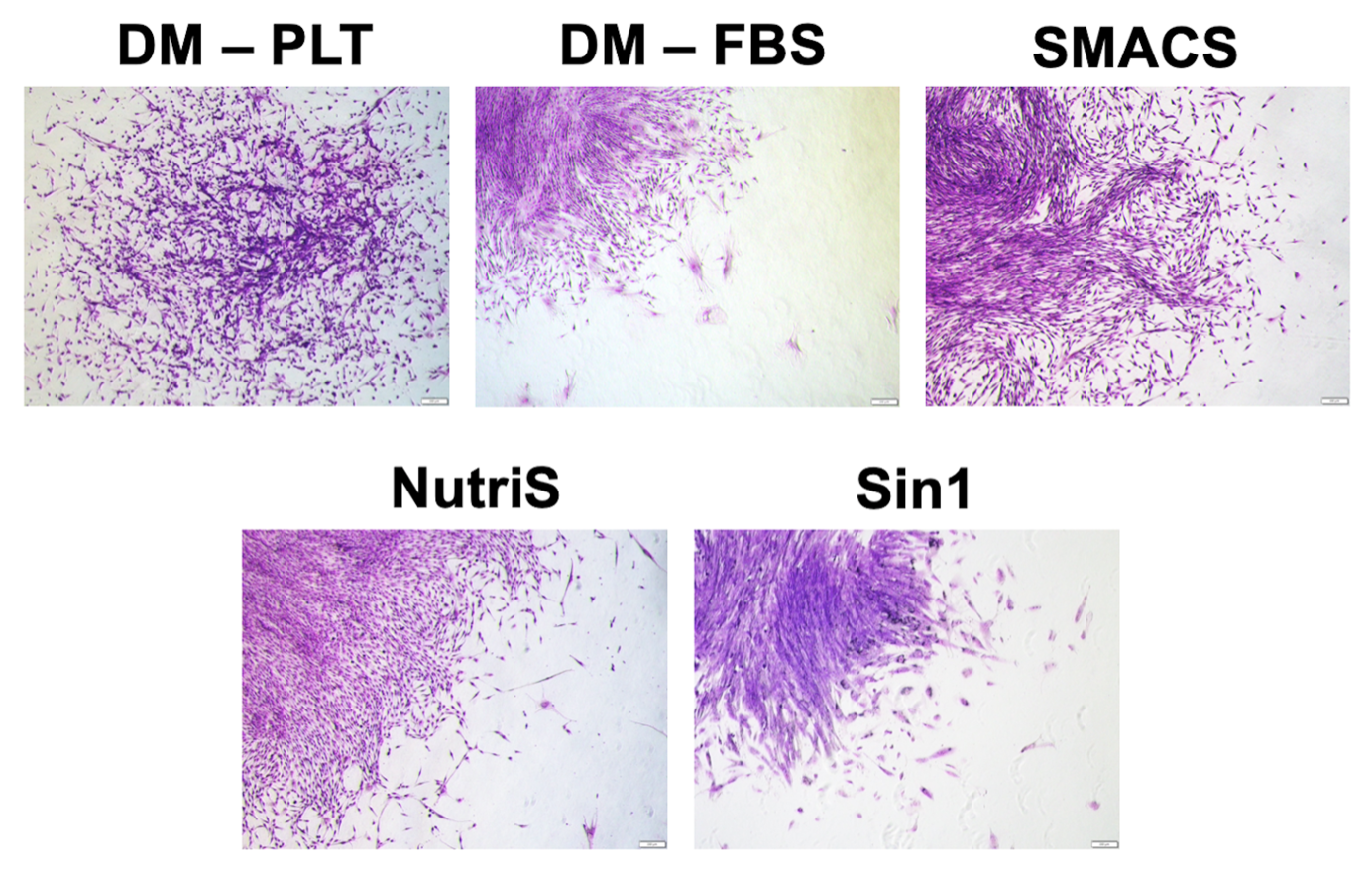

Supplement: S3 Fig — Sin1, NutriS, DM-PLT, SMACS, DM-FBS are UCMSCs cultured in STEMin1, NutriStem, DMEM/F12 medium supplemented with 5% PLT, StemMACS and DMEM/F12 medium supplemented with 10% FBS, respectively. (TIF) [file pone.0313697.s003.tif]

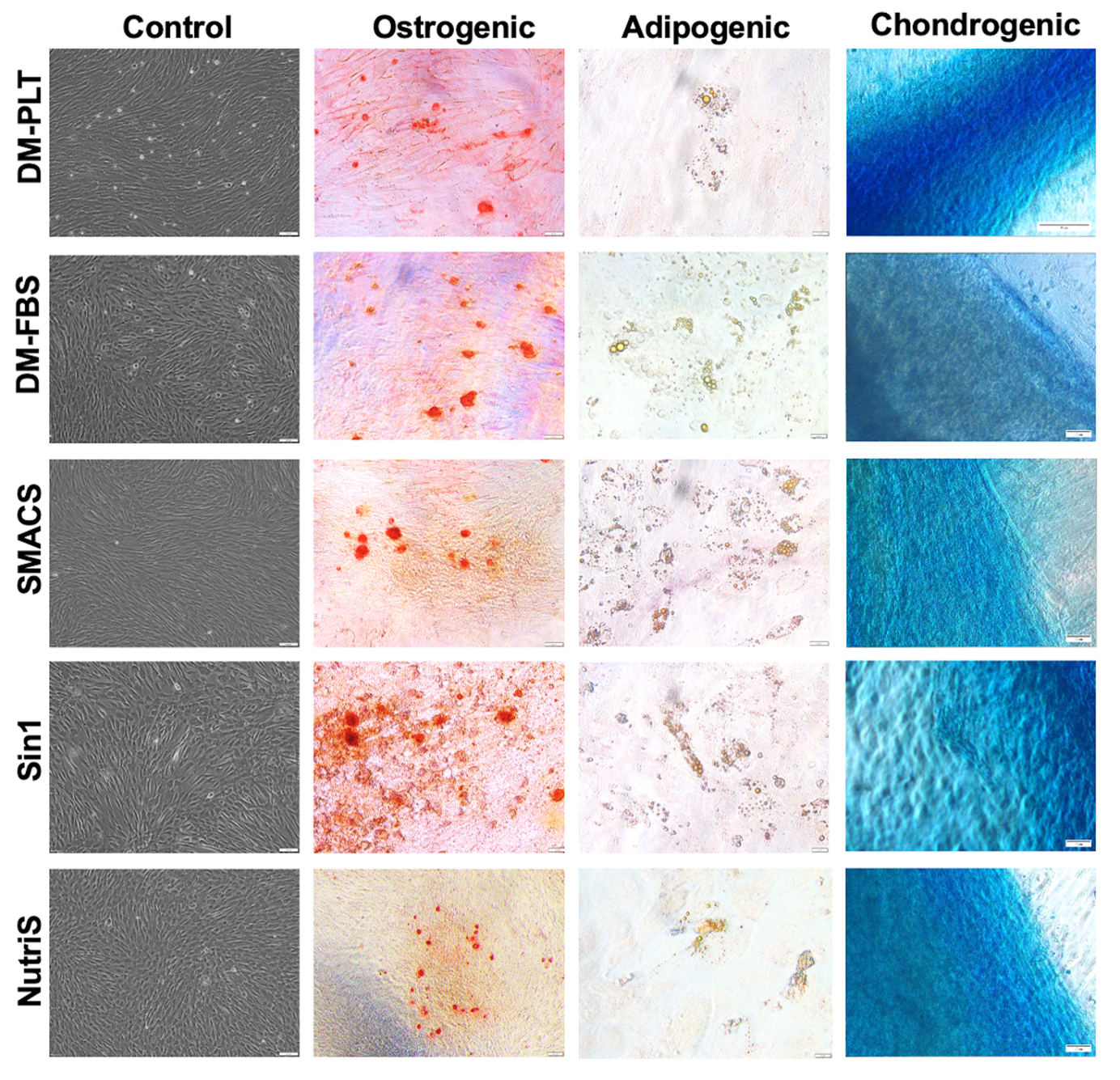

Supplement: S4 Fig — MSCs were cultured in osteogenic, adipogenic, and chondrogenic induction media and stained with corresponding staining dyes. Sin1, NutriS, DM-PLT, SMACS, DM-FBS are UCMSCs cultured in STEMin1, NutriStem, DMEM/F12 medium supplemented with 5% PLT, StemMACS and DMEM/F12 medium supplemented with 10% FBS, respectively. (TIF) [file pone.0313697.s004.tif]

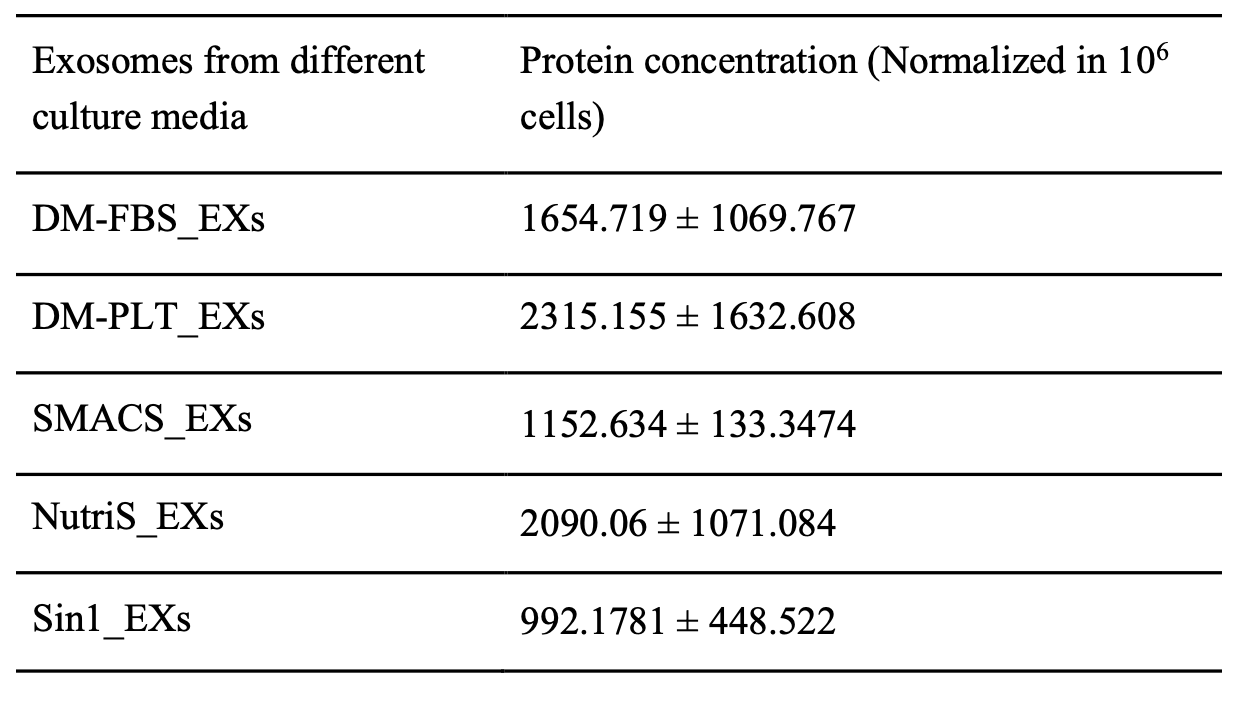

Supplement: S5 Fig — DM-FBS_EXs, DM-PLT_EXs, SMACS_EXs, NutriS_EXs, Sin1_EXs are EXs from UCMSCs cultured in DMEM/12 + 10% FBS, DMEM/F12 + 5% PLT, StemMACS, NutriStem, and STEMin1, respectively. (TIF) [file pone.0313697.s005.tif]

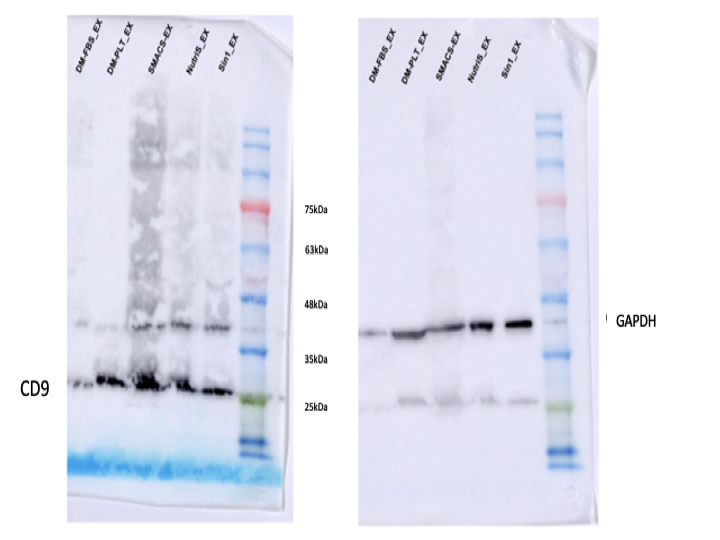

Supplement: S6 Fig — DM-FBS_EXs, DM-PLT_EXs, SMACS_EXs, NutriS_EXs, Sin1_EXs are EXs from UCMSCs cultured in DMEM/12 + 10% FBS, DMEM/F12 + 5% PLT, StemMACS, NutriStem, and STEMin1, respectively. (TIF) [file pone.0313697.s006.tif]

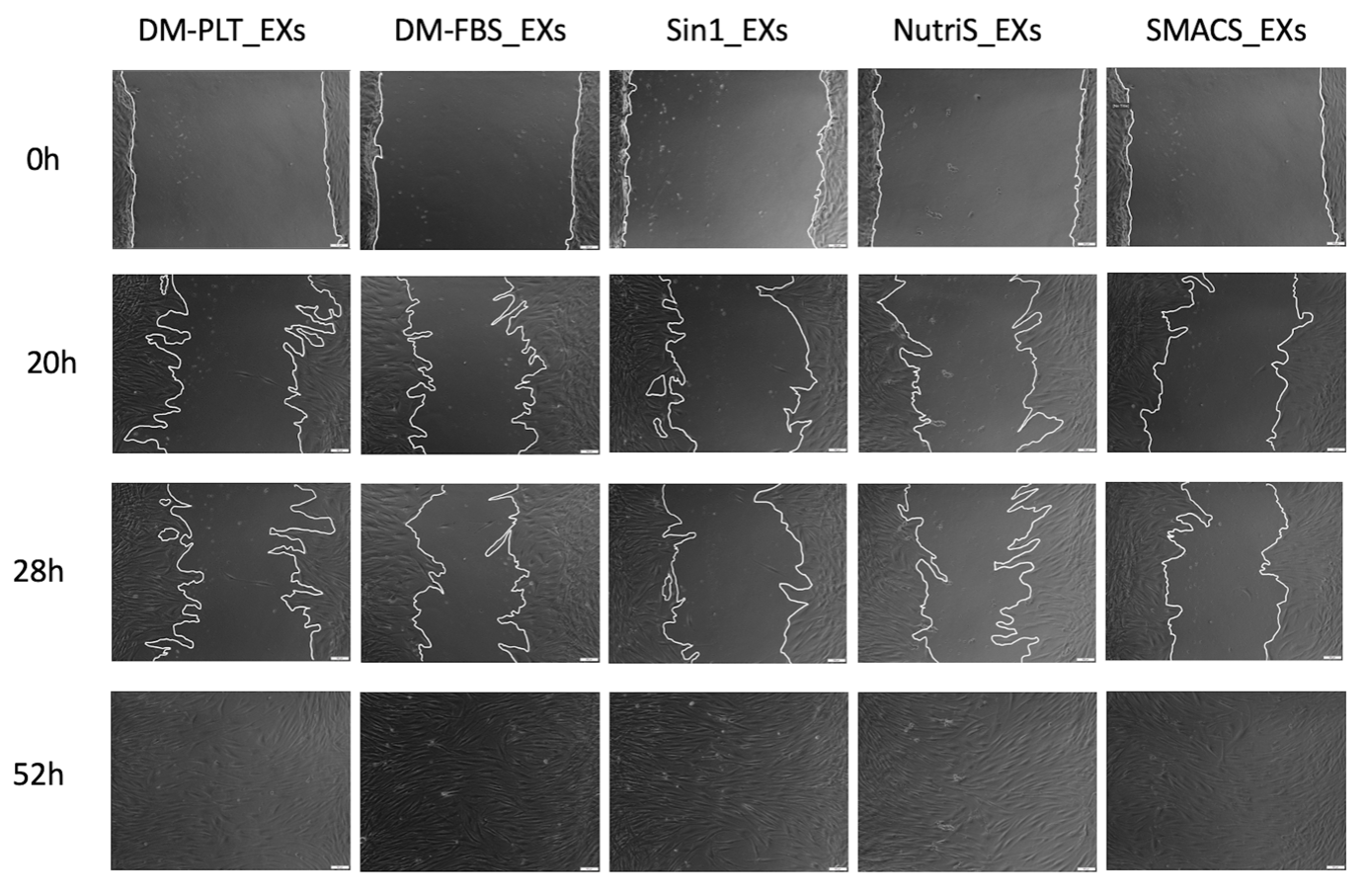

Supplement: S7 Fig — DM-FBS_EXs, DM-PLT_EXs, SMACS_EXs, NutriS_EXs, Sin1_EXs are EXs from UCMSCs cultured in DMEM/12 + 10% FBS, DMEM/F12 + 5% PLT, StemMACS, NutriStem, and STEMin1, respectively. (TIF) [file pone.0313697.s007.tif]
